# Clinical benefit of AI-assisted lung ultrasound in a resource-limited intensive care unit

**DOI:** 10.1186/s13054-023-04548-w

**Published:** 2023-07-01

**Authors:** Phung Tran Huy Nhat, Nguyen Van Hao, Phan Vinh Tho, Hamideh Kerdegari, Luigi Pisani, Le Ngoc Minh Thu, Le Thanh Phuong, Ha Thi Hai Duong, Duong Bich Thuy, Angela McBride, Miguel Xochicale, Marcus J. Schultz, Reza Razavi, Andrew P. King, Louise Thwaites, Nguyen Van Vinh Chau, Sophie Yacoub, VITAL Consortium, Dang Phuong Thao, Dang Trung Kien, Doan Bui Xuan Thy, Dong Huu Khanh Trinh, Du Hong Duc, Ronald Geskus, Ho Bich Hai, Ho Quang Chanh, Ho Van Hien, Huynh Trung Trieu, Evelyne Kestelyn, Lam Minh Yen, Le Dinh Van Khoa, Le Thanh Phuong, Le Thuy Thuy Khanh, Luu Hoai Bao Tran, Luu Phuoc An, Angela Mcbride, Nguyen Lam Vuong, Nguyen Quang Huy, Nguyen Than Ha Quyen, Nguyen Thanh Ngoc, Nguyen Thi Giang, Nguyen Thi Diem Trinh, Nguyen Thi Le Thanh, Nguyen Thi Phuong Dung, Nguyen Thi Phuong Thao, Ninh Thi Thanh Van, Pham Tieu Kieu, Phan Nguyen Quoc Khanh, Phung Khanh Lam, Phung Tran Huy Nhat, Guy Thwaites, Louise Thwaites, Tran Minh Duc, Trinh Manh Hung, Hugo Turner, Jennifer Ilo Van Nuil, Vo Tan Hoang, Vu Ngo Thanh Huyen, Sophie Yacoub, Cao Thi Tam, Duong Bich Thuy, Ha Thi Hai Duong, Ho Dang Trung Nghia, Le Buu Chau, Le Mau Toan, Le Ngoc Minh Thu, Le Thi Mai Thao, Luong Thi Hue Tai, Nguyen Hoan Phu, Nguyen Quoc Viet, Nguyen Thanh Dung, Nguyen Thanh Nguyen, Nguyen Thanh Phong, Nguyen Thi Kim Anh, Nguyen Van Hao, Nguyen Van Thanh Duoc, Pham Kieu Nguyet Oanh, Phan Thi Hong Van, Phan Tu Qui, Phan Vinh Tho, Truong Thi Phuong Thao, Natasha Ali, David Clifton, Mike English, Jannis Hagenah, Ping Lu, Jacob McKnight, Chris Paton, Tingting Zhu, Pantelis Georgiou, Bernard Hernandez Perez, Kerri Hill-Cawthorne, Alison Holmes, Stefan Karolcik, Damien Ming, Nicolas Moser, Jesus Rodriguez Manzano, Liane Canas, Alberto Gomez, Hamideh Kerdegari, Andrew King, Marc Modat, Reza Razavi, Miguel Xochicale, Walter Karlen, Linda Denehy, Thomas Rollinson, Luigi Pisani, Marcus Schultz, Alberto Gomez

**Affiliations:** 1 Oxford University Clinical Research Unit, Ho Chi Minh City, Vietnam; 2 School of Biomedical Engineering Imaging Sciences, King’s College London, London, UK; 3 Mahidol Oxford Tropical Medicine Research Unit, Bangkok, Thailand; 4 Hospital of Tropical Diseases, Ho Chi Minh City, Vietnam; 5 University of Medicine and Pharmacy, Ho Chi Minh City, Vietnam; 6 Centre for Tropical Medicine and Global Health, University of Oxford, Oxford, UK

**Keywords:** Lung ultrasound, Intensive care unit, Real-time, Artificial intelligence, Deep learning

## Abstract

**Background:**

Interpreting point-of-care lung ultrasound (LUS) images from intensive care unit (ICU) patients can be challenging, especially in low- and middle- income countries (LMICs) where there is limited training available. Despite recent advances in the use of Artificial Intelligence (AI) to automate many ultrasound imaging analysis tasks, no AI-enabled LUS solutions have been proven to be clinically useful in ICUs, and specifically in LMICs. Therefore, we developed an AI solution that assists LUS practitioners and assessed its usefulness in  a low resource ICU.

**Methods:**

This was a three-phase prospective study. In the first phase, the performance of four different clinical user groups in interpreting LUS clips was assessed. In the second phase, the performance of 57 non-expert clinicians with and without the aid of a bespoke AI tool for LUS interpretation was assessed in retrospective offline clips. In the third phase, we conducted a prospective study in the ICU where 14 clinicians were asked to carry out LUS examinations in 7 patients with and without our AI tool and we interviewed the clinicians regarding the usability of the AI tool.

**Results:**

The average accuracy of beginners’ LUS interpretation was 68.7% [95% CI 66.8–70.7%] compared to 72.2% [95% CI 70.0–75.6%] in intermediate, and 73.4% [95% CI 62.2–87.8%] in advanced users. Experts had an average accuracy of 95.0% [95% CI 88.2–100.0%], which was significantly better than beginners, intermediate and advanced users (*p* < 0.001). When supported by our AI tool for interpreting retrospectively acquired clips, the non-expert clinicians improved their performance from an average of 68.9% [95% CI 65.6–73.9%] to 82.9% [95% CI 79.1–86.7%], (*p* < 0.001). In prospective real-time testing, non-expert clinicians improved their baseline performance from 68.1% [95% CI 57.9–78.2%] to 93.4% [95% CI 89.0–97.8%], (*p* < 0.001) when using our AI tool. The time-to-interpret clips improved from a median of 12.1 s (IQR 8.5–20.6) to 5.0 s (IQR 3.5–8.8), (*p* < 0.001) and clinicians’ median confidence level improved from 3 out of 4 to 4 out of 4 when using our AI tool.

**Conclusions:**

AI-assisted LUS can help non-expert clinicians in an LMIC ICU improve their performance in interpreting LUS features more accurately, more quickly and more confidently.

**Supplementary Information:**

The online version contains supplementary material available at 10.1186/s13054-023-04548-w.

## Background

In recent years, point-of-care ultrasound (POCUS) has proved to be a useful bedside imaging technique for the assessment of critically ill patients for both diagnosis and therapeutic management [1–3]. LUS does not expose patients to radiation and has been shown to be more sensitive and specific in the diagnosis of many pulmonary pathologies when compared to chest x-ray (CXR) [4], hence the potential for application of LUS in low- and middle-income countries (LMICs) is high [5, 6].

Respiratory failure due to infectious disease is one the most common reasons for ICU admission in LMICs, for example, due to dengue, sepsis, or malaria and more recently Covid-19. In severe cases, progression to Acute Respiratory Distress Syndrome (ARDS) can occur, which has a high mortality, and leaves survivors with significant pulmonary morbidity [7–9]. LUS protocols such as the BLUE protocol and the FALLS protocol [10] are designed to assist doctors with the diagnosis and management of pulmonary and cardiac conditions. The Kigali modification of the Berlin ARDS criteria [11] has helped to diagnose ARDS in resource-limited settings by using ultrasound instead of CXR or Computed Tomography (CT) which are often unavailable in these settings. However, LUS is operator-dependent and requires extensive training for image acquisition and interpretation. The lack of qualified ultrasound professionals and the paucity of training programs are significant obstacles to the implementation of LUS in LMIC ICUs.

Artificial intelligence (AI), particularly deep learning, has made substantial advances in ultrasound imaging analysis during the last decade. For LUS, most existing work is limited to AI-recognition of a single artefact (B-lines), or, more recently, multi-class classification for a specific lung disease, focusing on COVID-19 [12–17]. Still, the implementation and validation of developed algorithms in clinical settings remains very limited and is focused mainly on fetal and cardiac ultrasound [18–20].

In the case of LUS there are, to date, no published investigations on real-time deployment of AI-enabled tools to recognise multiple LUS patterns. In addition to improving accuracy, automatic recognition can help improve confidence in, and reduce the time spent by the clinician on, image interpretation, especially where resources are limited [21].

For these reasons, our study aimed to evaluate a real-time AI-ultrasound system designed to detect five common LUS patterns relevant to LMIC ICU clinicians. Building upon our previous AI methodology work, in this study, we evaluated the clinical need and assessed the utility of AI-enabled LUS in an ideal offline scenario followed by real-time clinical practice.

## Methods

Our study was carried out in Vietnam in three phases. In the first phase we confirmed the use-case for our tool and set a minimum performance target for the AI system. This was an online interactive survey of participants from multiple hospitals in Vietnam. The second and third phases assessed the use of the AI tool and were carried out at the Hospital for Tropical Diseases (HTD) Ho Chi Minh City. In this study we deliberately chose to focus on the non-expert clinician in phase 2 and 3, as there are currently few experts or advanced users, and inexperienced clinicians are the target users for our tool. The study was approved by the Scientific and Ethical Committee of the Hospital for Tropical Diseases and the Oxford Tropical Research Ethics Committee. All participants gave written informed consent.

### Phase 1: baseline characterization of user performance in LUS interpretation without AI support

An online survey was completed by 276 participants of an online LUS training course attended by doctors from multiple centres around Vietnam on September 4th, 2021. The level of expertise was self-assessed by clinicians using four pre-defined categories: (1) beginners, defined as “just know about LUS but have not practiced in patients”, (2) intermediate, a clinician who has been carrying out LUS (< 2 times/week) but have not used the findings for clinical assessment, (3) advanced, a clinician who uses LUS in daily practice and its findings are used for clinical assessment, and (4) expert, a clinician who specialised in lung ultrasound and has more than 5 years’ experience. The participants were asked to identify the findings in a series of 10 LUS clips from adult patients hospitalized with dengue shock and septic shock displaying A-lines, B-lines, Confluent B-lines, Consolidation and Pleural Effusion (2 of each) given in a set order (samples are shown in the Additional file 1: Fig. S1). Responses were compared with the expert-defined labels consistent with our data curation process. All clips were sourced and labelled with agreement by three ultrasound-trained clinicians and one expert.

### Phase 2: development and clinical validation of the real-time AI-assisted LUS framework (RAILUS) in controlled environment

In this phase, we evaluated the impact of our bespoke LUS AI system on clinicians’ performance in a controlled environment, using a set of images already obtained by LUS experts. Our RAILUS (Real-time AI-assisted LUS) system is described in greater detail in the Additional file 1 (Table S1, Table S2, Fig. S2, Fig. S3) and consists of an AI model integrated into the PRETUS platform (Fig. S4) [22].

**Fig. 1:**
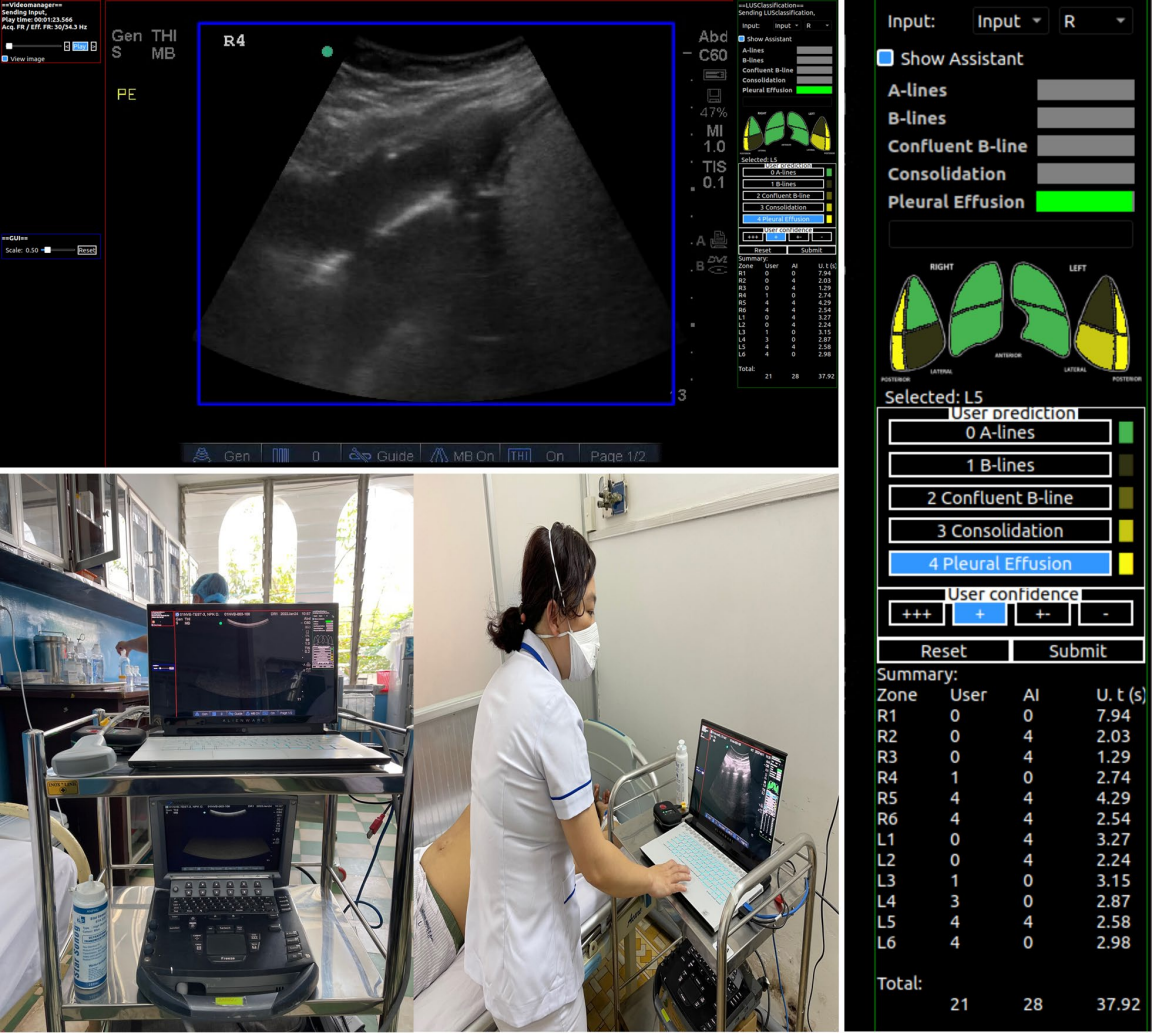
Real-time AI-assisted LUS framework (RAILUS)

Briefly, the system provides continuous real-time prediction through a laptop and can be used in both pre-recorded LUS clips and the real-time clinical environment with clinicians carrying out the LUS examination, with the ultrasound machine’s video output connected to the laptop (Fig. 1). RAILUS also captures the user prediction, model prediction, time-to-interpret, and the confidence of the users, and can be used in any ultrasound machine with a video output port.

We evaluated the performance of RAILUS in a controlled environment in workshops for 57 non-expert clinicians in three different clinical settings (tertiary referral centre, COVID-19 field hospital and academic ventilation training course). Participants were given 1 h of training to become familiar with the RAILUS software then asked to interpret LUS videos (as in phase 1) firstly, without the RAILUS tool and then with the RAILUS tool (participants were blinded to the AI-assisted interpretation). Reponses were used to calculate the average accuracy of the interpretation against expert-defined labels.

### Phase 3: real-time implementation of RAILUS software in critically ill patients

Real-time evaluation was carried out prospectively by a subset of non-expert clinicians from Phase 2 who carried out LUS with and without the AI system in the Emergency Department or on ICU patients at the Hospital for Tropical Diseases Ho Chi Minh City. Eligible patients were adults aged ≥ 18 years with dengue shock admitted between December 2021 to February 2022. Patients who were allergic to ultrasound gel or had open wounds on their chest were excluded. This study was approved by the Oxford Tropical Research Ethics Committee (OxTREC) and the HTD Institutional Review Board. Patients underwent LUS scans by non-expert clinicians who had received 1 h of training similar to above. Participating clinicians were randomly assigned to perform the LUS scans on patients following a standard 12 zone LUS protocol with or without the RAILUS software (Additional file 1: Fig. S5). When assigned to the non-AI group, the AI-assisted interpretation was turned off. A LUS expert then performed the same 12 zone scan on the same patient within 2 h of the non-expert clinicians performing their LUS scans. To assess whether each scan was of adequate diagnostic quality, we performed an independent expert validation (blinded to whether the study was performed using the AI tool or not). To evaluate usability, a questionnaire was administered to the clinicians at the end of the procedure. The full questionnaire can be found in Additional file 1: Table S4 and Table S5.

Results were evaluated to quantify the accuracy of the interpretation against the expert, the time required to interpret single lung zones, and the clinicians’ perceived confidence in their interpretation (from 1 to 4. with 1 = not confident and 4 = very confident).

### Statistical analysis

To assess the performance of the clip classification task, we assessed (1) overall accuracy, calculated as the number of correctly classified clips as a fraction of the total number of clips; (2) average accuracy, calculated as the average over all lung pathologies of per-pathology accuracy; (3) F-score; (4) precision and (5) sensitivity (recall). Confusion matrices were calculated and reported. The discriminative variable of demographic information was reported as a percentage. To compare the clinicians’ performance with and without our AI tool, we utilized confusion matrices with the horizontal axis and vertical axis corresponding to predicted label and true label, respectively. The proportion of clips that were accurately classified are reported with 95% confidence intervals (95% CI).

## Results

### User performance in LUS interpretation without AI support (phase 1)

The demographic information of the clinicians and principal challenges encountered are provided in Additional file 1: Tables S6 and S7. The majority (194/276, 70%) of clinicians identified themselves as beginners, and there were very few experts (4/276, 1%). Most clinicians identified “image interpretation" as the main challenge.

**Fig. 2:**
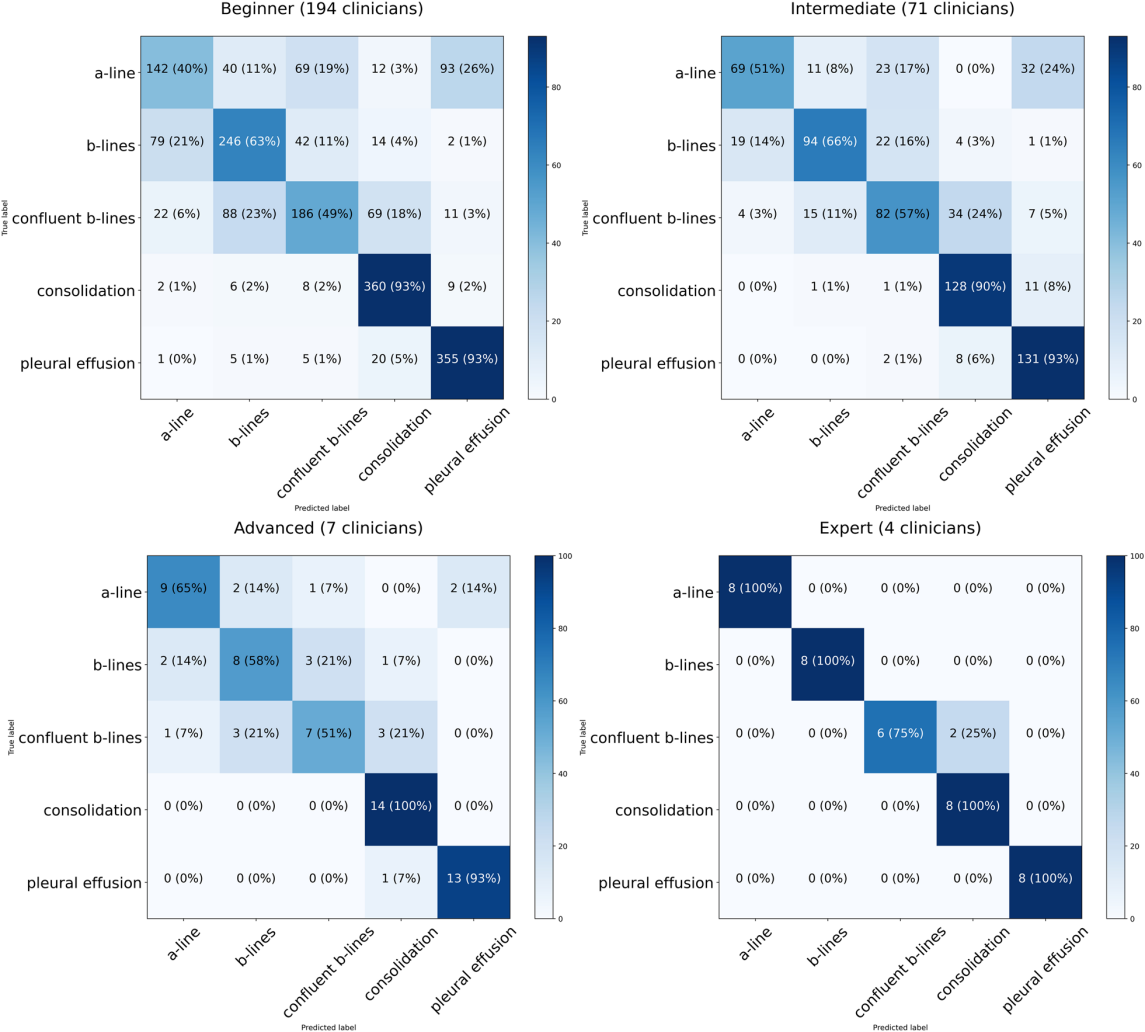
The results of the manual video classification of clinicians with 4 levels of expertise

The unassisted video classification results are shown in the confusion matrices in Fig. 2 (showing total count and percentage). Experts showed excellent ability to accurately classify LUS clips. For all other clinician categories, there was more difficulty in differentiating A-lines, B-lines and confluent B-lines, and relative ease in identifying consolidation and pleural effusion.

Horizontal and vertical axis show predicted label and expert-defined label, respectively. The numbers in each cell indicate total count (percentage of total). Cells are colored by percentage.

Interestingly, although the average accuracy was correlated to the level of expertise (beginners: 68.7% (95% CI 66.8–70.7%), intermediate 72.2% (95% CI 70.0–75.6%), advanced 73.4% (95% CI 62.2–87.8%), and experts 95.0% (95% CI 88.2–100.0%)), (p < 0.001), the difference in performance between beginners, intermediate and advanced users was relatively small (< 5%).

### Performance and clinical validation of the real-time AI-assisted LUS framework (RAILUS) in  a controlled environment (phase 2)

**Fig. 3:**
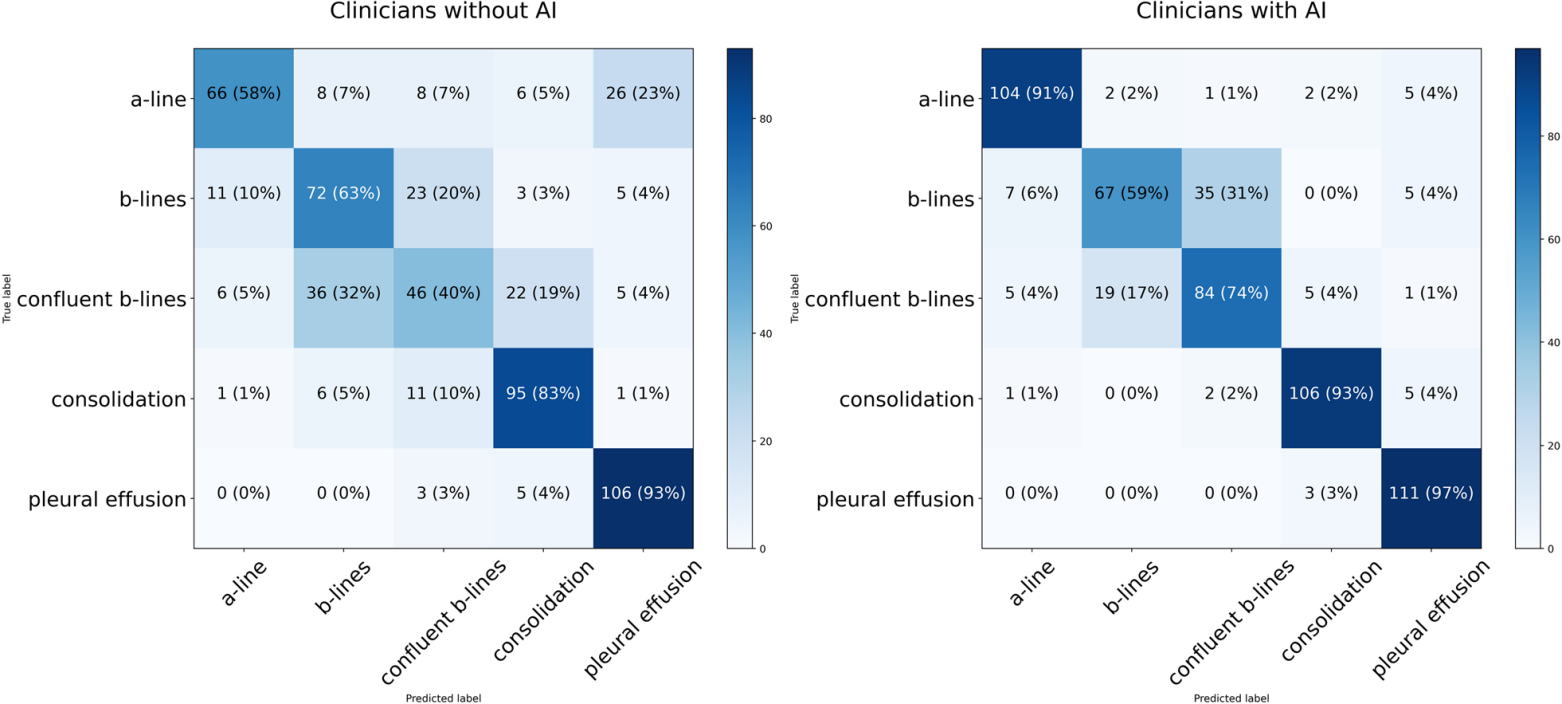
Confusion matrices of clinicians with, and without RAILUS in a controlled environment using expert-acquired clips

The performance of the proposed AI model is shown in the Additional file 1: Table S2 and Fig. S3. The demographic information of the 57 non-expert clinicians is provided in the Additional file 1: Tables S6. The performance of clinicians in identifying the LUS clips presented to them was improved using the RAILUS software, with a mean accuracy of 82.9% (95% CI 86.7–79.1%), compared to 68.9% (95% CI 65.6–73.9%), (*p* < 0.001) (Fig. 3). The accuracy of all classes increased significantly except for the case of B-lines classification, which reduced from 63% to 59% when using RAILUS. The performance of the subset of 14 clinicians who participated in both phase 2 and phase 3 was shown in the Additional file 1: Fig. S6.

### Real-time implementation of RAILUS software in critically ill patients (phase 3)

In total, seven patients with dengue shock were recruited for real-time testing of the RAILUS software. Additional file 1: Table S3 and Table S6 show the characteristics of the patients and the 14 non-expert clinicians, respectively. Overall, 26 LUS exams were carried out, resulting in 168 LUS videos (4 s each) performed with the AI tool and 144 LUS videos performed without the AI tool. Image quality was acceptable for all scans. No adverse events occurred during scanning.

**Fig. 4:**
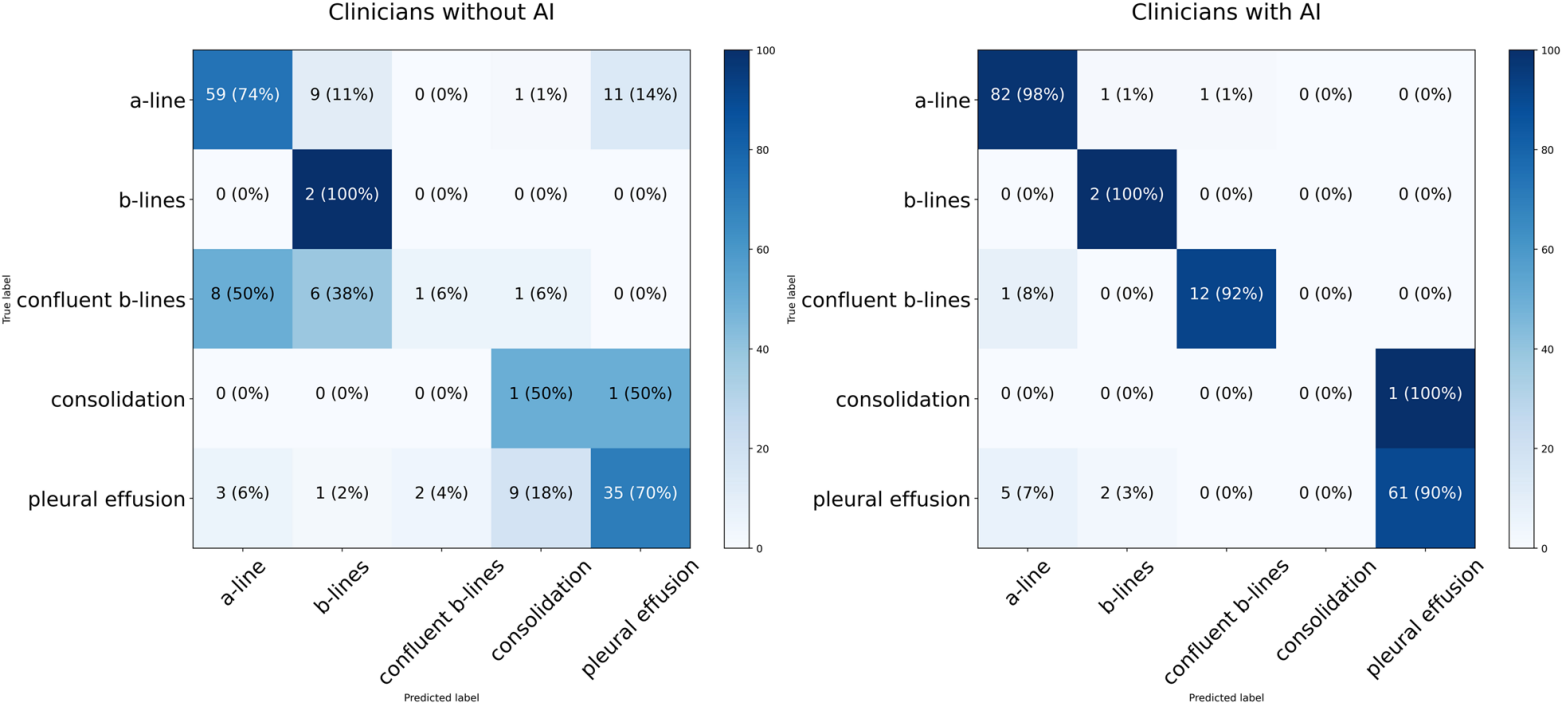
Confusion matrices of clinicians with and without RAILUS in real-time

Accuracy of image identification was higher in those using the RAILUS AI tool than those using the standard LUS technique: 93.4% (95% CI 89.0–97.8%) compared to 68.1% (95% CI 57.9–78.2%), (*p* < 0.001). Performance was better in all classes for clinicians using our AI tool compared to those without AI assistance as shown in Fig. 4. In particular, A-line detection accuracy rose from 74 to 98%, and Confluent B-line detection accuracy rose from 6 to 92%.

The time taken to interpret one LUS clip was shorter when using the RAILUS software compared to the standard LUS technique: a median of 5.0 s (IQR 3.5–8.8) compared to 12.1 s (IQR 8.5–20.6) (*p* < 0.001). In addition, the median confidence level of clinicians improved from 3 out of 4 to 4 out of 4 when scanning patients using the AI tool.

Overall, most operators found the tool usable, useful, and beneficial. Usability questionnaires showed an overall positive impression of the RAILUS tool. Most clinicians (13/14, 93%) found the AI-assisted tool useful in the clinical context and wanted to use the tool in the future (12/14, 86%). 64% (9/14) of clinicians thought the tool was useful for both real-time and post-exam evaluation of LUS imaging while only 7% (1/14) thought it was only useful for post-exam evaluation. Interestingly, 71% (10/14) of clinicians wanted the radiologist/expert to re-evaluate their interpretation with the AI tool. Moreover, 64% (9/14) of clinicians felt more confident when performing LUS with the AI tool, compared to only 7% (1/14) being most confident without the AI tool.

Regarding the concerns of clinicians, the issues of legal responsibility (9/14, 64%) and data privacy (4/14, 28%) were identified. More details about the usability survey can be found in the Additional file 1: Figure S7.

## Discussion

In this paper, we have evaluated an AI-assisted LUS system in a resource-limited ICU setting. To this end, we initially assessed the baseline performance of clinicians in a LUS image classification task. Our results show that there is a significant gap between beginners, intermediate, advanced clinicians, and experts in LUS interpretation, particularly in B line interpretation. This is consistent with our survey where the majority of the participants stated that image interpretation was their most significant challenge in performing LUS. This performance gap and the challenging nature of this task may prevent non-expert clinicians from carrying out LUS examinations in practice. In low resource settings like Vietnam, there are very few experts or even advanced clinicians in LUS. As our survey in phase 1 showed, across Vietnam, there were very few advanced users or experts, hence the need for our tool. Even in our setting for phase 3 of this study—a large teaching hospital- there are no experts in LUS. Developing an AI tool that can assist inexperienced users in this setting could be highly beneficial for patient outcomes and improving quality of care.

A crucial aim of our study was to investigate whether operators improved their baseline performance when assisted by our RAILUS AI system. Our study showed that performance improved by 15% when using RAILUS in a controlled environment (with expert-obtained clips), but by 25% when using RAILUS prospectively in real-time. Notably, this represented a level exceeding the baseline set by advanced clinicians in Phase 1. In addition, interpretation was approximately twice as fast when using the AI system. Of note, the clinicians using the tool were those already involved in routine care of these patients (imaging staff, infectious disease doctors and ICU staff) but they still showed a significant improvement in performance with the AI tool in both phases 2 and 3. The performance of clinicians in interpreting B-lines reduced slightly in the second phase. We note that this is mainly due to difficulty distinguishing B lines from confluent B lines. Our User Interface (UI) allowed several possibilities to be simultaneously displayed, and commonly this meant that both B line and confluent B lines were predicted for the same clips (although with varying degrees of certainty as represented by the green line in the UI—Additional File 1: Figure S4). The ultimate decision was left with the clinician, and hence this introduces interesting questions about trust in AI and clinical decision making. In phase 3 there were only 2 loops with B-lines, thus a small sample size from which to make definite conclusions in this sub-sample, and also we cannot exclude other contextual influences on decision making. In future studies, sample size calculations should take the incidence rate of each lung pattern into account and also make efforts to understand better clinicians' reasons for following AI predictions (or not).

Finally, these quantitative results were supported by the post-experiment surveys, which revealed that the AI-assisted tool was felt to be useful in the clinical context and most clinicians confirmed they were keen to use the tool in the future. However, the concerns raised about data privacy and legal responsibility when using an AI-assisted tool are valid. As the application of AI in ultrasound is in its infancy, there are relatively few regulations on how to legally implement it in routine healthcare practice or who will be response for AI derived medical errors, particularly in low resource settings. By improving the accuracy, speed and confidence of bedside LUS, it might help clinicians in ICUs in LMICs to better manage critically ill patients with various lung pathologies. This could especially benefit the monitoring of patients during fluid resuscitation where fluid balance is critical to achieve a stable haemodynamic status without causing fluid overload e.g., pulmonary oedema.

We believe our study represents an important step towards real-time implementation of AI in LMIC ICUs, but nevertheless has some limitations. The dataset used is from patients with severe dengue or sepsis so it remains to be seen how these results would translate to patients with other diseases. Furthermore, while we have designed our system to be agnostic to ultrasound devices, clinical validation of the tool on other types of device (such as portable devices) is yet to be performed. Our study focused on the individual findings of LUS. Clinical practice requires a more nuanced interpretation of these findings for optimal benefit, for example, whether the confluent B-lines and consolidations are focal or non-focal to discriminate between pneumonia or pulmonary edema. For the first phase, the order of questions in the survey was not randomized but it was distributed electronically to a pool of participants distributed across Vietnam who could not share the survey with each other. In terms of clinical validation, the study did not randomize the patients to either use the AI tool or not. Future studies should explore the potential benefits of AI tools for advanced or expert users, the regulatory, ethical and cultural issues of the clinical use of AI methods in different healthcare settings. In addition, further technical development including but not limited to AI interpretability, AI fairness, quantification of pleural effusion, or more complex LUS patterns such as pneumothorax can be attractive fields of research.

## Conclusions

This is the first study of real-time implementation of AI-assisted LUS interpretation in critically ill patients, demonstrating the feasibility of our system for non-expert clinicians, with limited LUS experience, to acquire and interpret lung ultrasound in critically ill patients.

## Supplementary Information

**Additional file 1**: . Model development and evaluation, description of RAILUS software and usability questionnaire.

## Data Availability

The datasets used and/or analysed during the current study are available from the corresponding author on reasonable request. The codes and model weights needed to deploy the RAILUS tool for the study are available at https://github.com/vital-ultrasound/public-lung.
