# Supplementary material for: Clinical benefit of AI-assisted lung ultrasound in a resource-limited intensive care unit
Source: Crit Care. 2023 Jul 1;27:257. doi: 10.1186/s13054-023-04548-w (PMC10314555; doi:10.1186/s13054-023-04548-w)
Supplement: Supplementary file 1 — Additional file 1: . Model development and evaluation, description of RAILUS software and usability questionnaire. [file 13054_2023_4548_MOESM1_ESM.docx]

**Supplementary Appendix**

**This appendix is supplement to:** **Clinical benefit of AI–assisted lung ultrasound in a resource–limited ICU.**

Table of contents

Section 1. Design, Development and Performance of AI-assisted LUS video classification 3

Dataset 3

Model architecture 4

Performance AI-assisted LUS video classification algorithm 5

Section 2. Real-time AI-assisted LUS framework (RAILUS) framework 6

Section 3. Questionnaire - Usability of AI-assisted lung ultrasound interpretation tool 9

Part 1 – Demographics (10 questions) 9

Part 2 – Lung ultrasound video interpretation (10 standard videos and 10 videos with AI tool) 10

Part 3 – Usability (20 questions) 10

Reference 14

**Table of Figures and Tables**

Figure S1. Sample LUS image………………………………………………………………………………………3

Figure S2. Model architecture……………………………………………………………………………………….4

Table S1. Number of samples per class and training weight values of each class. …………………………………4

Table S2. Classification performance (F1-score, precision and recall) on the test set………………………………5

Figure S3. Confusion matrix of our proposed model………………………………………………………………..5

Figure S4. Real-time AI-assisted LUS framework (RAILUS)………………………………………………………6

Figure S5. Prospective study schema………………………………………………………………………………..8

Table S3. Baseline of characteristics of study patients……………………………………………………………...8

Figure S6. Performance of 14 clinicians in the third phase when using the tool offline in second phase…………..9

Table S4. Demographics of clinician’s questionnaires ……………………………………………………………..9

Table S5. Usability questionnaire…………………………………………………………………………………..10

Table S6. Demographics of the participants………………………………………………………………………..12

Figure. S7. Usability of the RAILUS software in real-time………………………………………………………...14

# Section 1. Design, Development and Performance of AI-assisted LUS video classification

## Dataset

In this study, the dataset was collected from 65 patients with dengue shock or septic shock admitted to the Hospital of Tropical Diseases (HTD) in Ho Chi Minh City, Vietnam between June 2019 and June 2020. The research was approved by the Oxford Tropical Research Ethics Committee (OxTREC) and the HTD Institutional Review Board. The dataset was split into training, validation and test as follows: 90% (3079 videos, four seconds each) was used for training and validation and the remaining 10% of the patients (322 videos, four seconds each) were left out for test. LUS examinations were carried out using a Sonosite M-Turbo machine (Fujifilm Sonosite, Inc., Bothell, WA) with a low medium frequency (3.5-5 MHz) convex probe by qualified sonographers. LUS was performed using a standardised operating procedure based on the Kigali ARDS protocol^1^: assessment for B-lines, consolidation and pleural effusion, performed at 6 points on each side of the chest (2 anterior, 2 lateral and 2 posterolateral).

These videos were annotated by expert sonographers using the VGG annotator tool^2^ . Five lung patterns were selected for multi-class classification, as introduced above and illustrated in Figure S1: A-line (normal lung), isolated B-lines, Confluent B-lines, Consolidation and Pleural effusion. The distribution of the overall data is shown in Table 3. Class imbalance was addressed during training by weighting each class contribution to the loss by the inverse of the number of samples in each class.


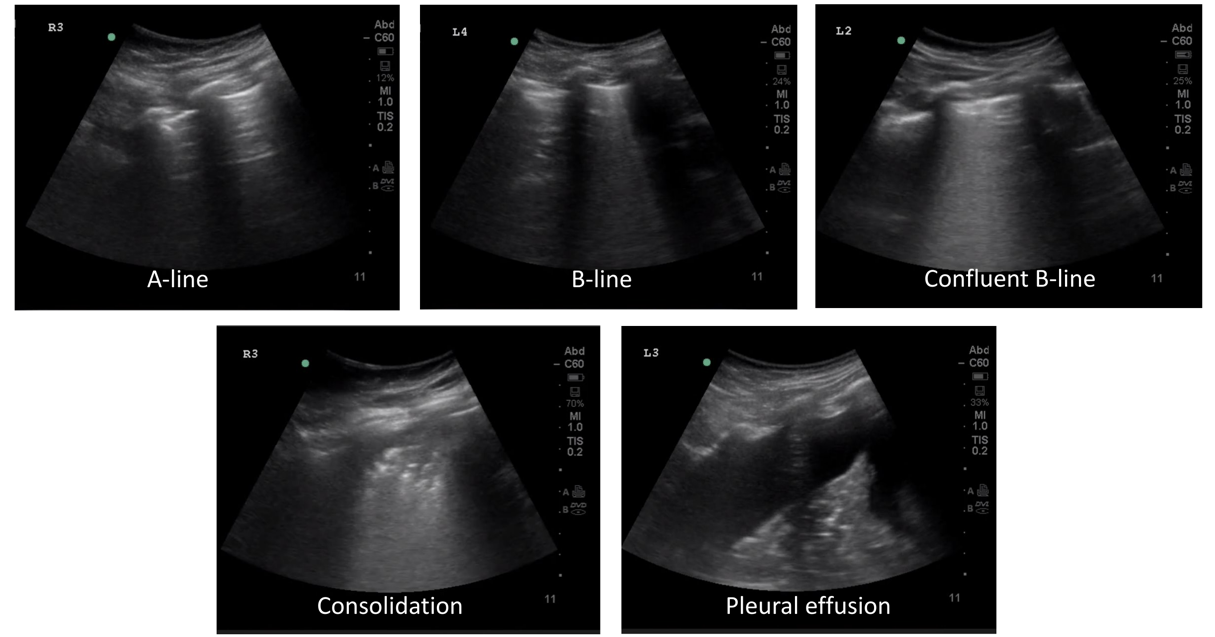


Fig. S1. Sample LUS images. The LUS artifacts reflect the ratio between air, lung tissue, fluid, or other biological components. A–line (representing a healthy lung, horizontal reverberation artifacts of the pleural line caused by multiple reflection), isolated B–lines (vertical hyperechoic artifacts deriving from the pleural line spreading to the edge of the screen, moving synchronously with the lung, more than 2 B–lines is considered abnormal), Confluent B–lines (many B–lines merge together and occupy a large area in the intercostal space, which is a sign of pulmonary interstitial syndrome), Consolidation (an echo–poor image juxtaposed to the pleural line (white arrow) and delimited by irregular boundaries, usually seen in pneumonia), Pleural effusion (hypoechoic space between the parietal and visceral pleura, caused by the build–up of excess fluid between the layers of the pleura outside the lungs due to heart failure, plasma leakage, pneumonia or pulmonary embolism).

AVI-format videos were cropped and masked to remove text and information outside of the scanning sector. The 640x480 pixel videos were downsampled using OpenCV into 64x64 pixels. For training, each four-second clip was converted into shorter clips of one second with an overlap of 20% between consecutive frames in the video.

## Model architecture

Our proposed model is an extension of the model from our previous work^3^ that was used for B-line classification and localization in LUS videos. The model architecture is depicted in Figure 3. It consists of three parts: a convolutional neural network (CNN) to extract frame-wise spatial features, a bidirectional long short-term memory (LSTM) network to extract temporal features from the video and a temporal attention mechanism to increase the weighting of frames that carry more useful information for the classification task. In this paper, we replaced the classification subnet, after the temporal attention mechanism, with a fully connected layer (with ReLU non-linear activation and dropout) and a 5-element final layer that produces a 1-hot 5D vector for 5 class classification.


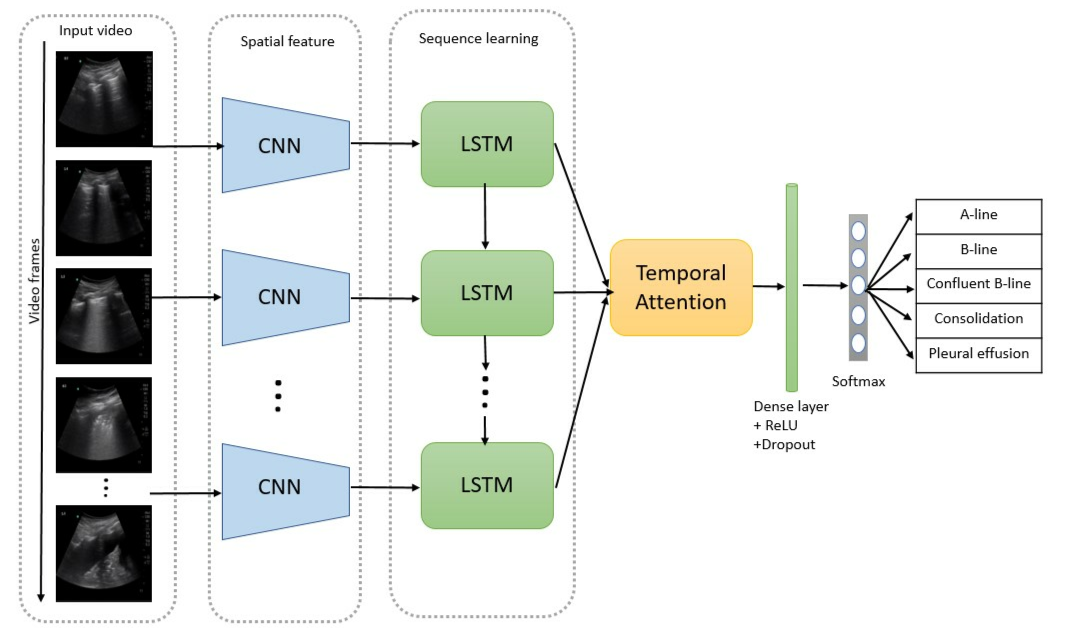


Figure S2. Model architecture

The model was implemented in Python 3 using the Keras library with a Tensorflow backend. It was trained using the Adam optimizer with a learning rate of 0·001. A batch size of 16 and batch normalization were applied for both CNN and LSTM network layers. Dropout of 0·2 and L2=10^-5^ for regularization were utilized. These parameters were found to give the best result on the validation set. LUS video data were augmented by adding horizontally-flipped frames to the training set. We used 5-fold cross validation and trained the network for 100 epochs. The trained models were evaluated on the independent test set.

## Performance of AI-assisted LUS video classification algorithm

The proposed AI model was trained with more than 3000 lung ultrasound videos from dengue and septic shock patients in HTD, acquired using a Sonosite M-Turbo (Fujifilm Sonosite, Inc., Bothell, WA) machine. The details of each class and the corresponding class weights (to balance the training) are shown in Table 4.5.

Table S1. Number of samples per class and training weight values of each class.

| Class | Number of videos | Weight |
| --- | --- | --- |
| A-line | 1825 | 1·0 |
| B-lines | 102 | 17·8 |
| Confluent B-lines | 138 | 11·4 |
| Consolidation | 21 | 78·3 |
| Pleural effusion | 993 | 1·6 |

Our model achieved an average accuracy of 81%±11%, with the test F1-scores for each class (A-line, B-lines, Confluent B-lines, Consolidation, Pleural Effusion) being 92%, 67%, 75%, 67%, and 90%, respectively. The relatively low accuracy for consolidation is due to consolidation being a relatively rare condition for which we have significantly less data, as shown in Table S1. Detailed results on F1 score, precision and recall are provided in Table S2.

Table S2. Classification performance (F1-score, precision and recall) on the test set

|  | F1 | Precision | Recall |
| --- | --- | --- | --- |
| A-line | 92% | 96% | 89% |
| B-line | 67% | 57% | 80% |
| Confluent B-lines | 75% | 68% | 83% |
| Consolidation | 67% | 75% | 60% |
| Pleural effusion | 90% | 87% | 93% |
| Average (±std) | 78%±11% | 77%±14% | 81%±11% |

To put the model performance in context, we provide the confusion matrix (in relative numbers) in Figure S3.


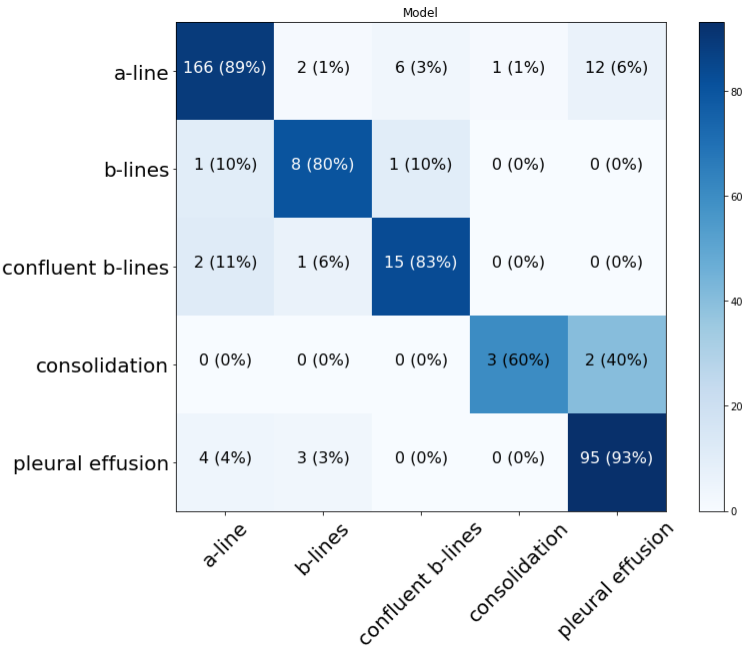


Figure S3. Confusion matrix of our proposed model

These results indicate clear trends supporting the suitability of our model to support clinicians. Our model outperforms beginners, intermediate and advanced users on average and in every class except for consolidation, for which we had significantly less data. Interestingly, all users have >90% accuracy in this class.

# Section 2. Real-time AI-assisted LUS framework (RAILUS) framework


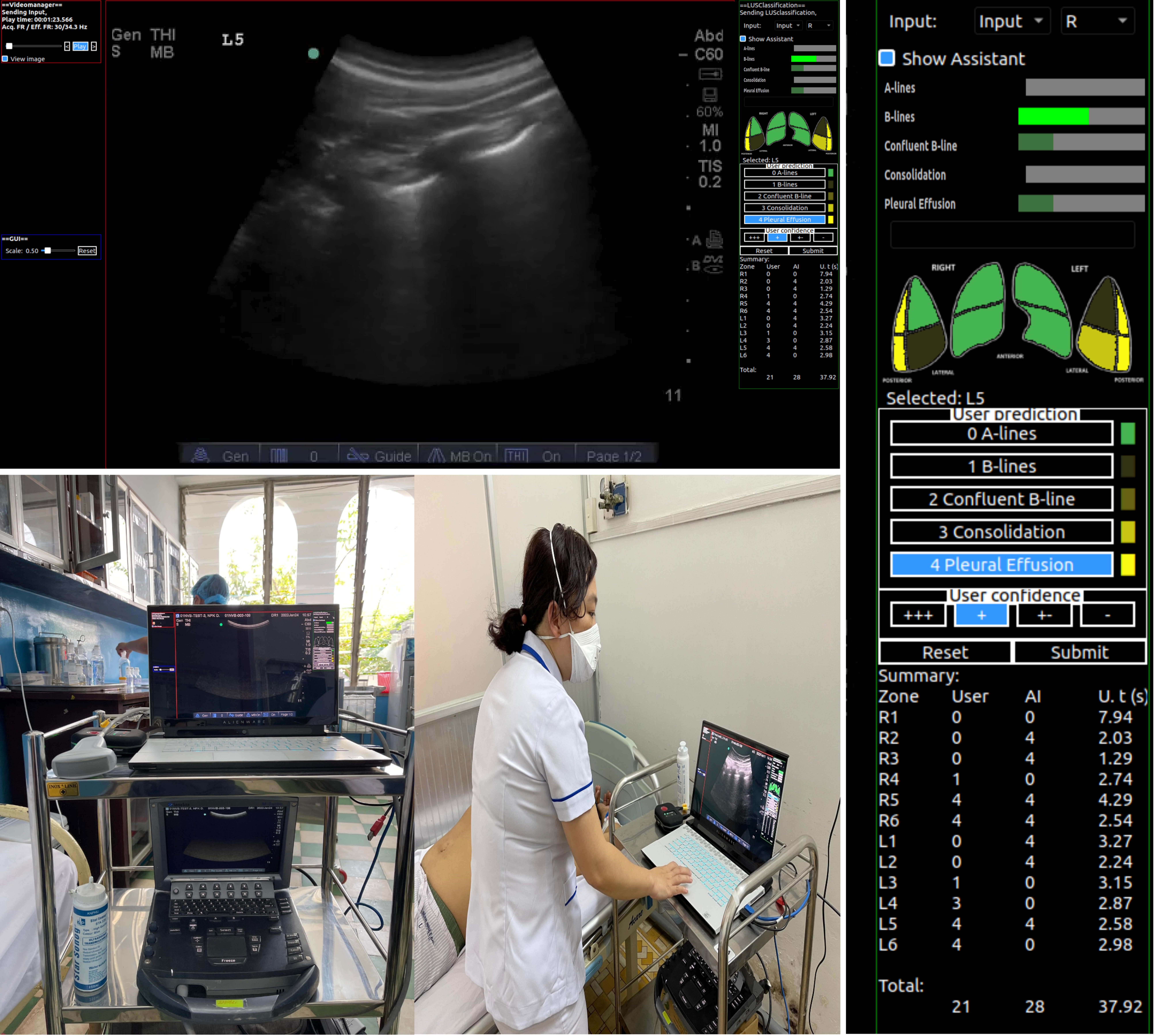


Figure S4. Real-time AI-assisted LUS framework (RAILUS)

The ultrasound machine HDMI output was connected with the laptop via a USB framegrabber. Instead of looking at the ultrasound machine screen, the user can look at the laptop’s screen. The code for RAILUS is made publicly available on a github repository (https://github.com/vital-ultrasound/public-lung)

On the left of the screen is the video resolution and scale. Depending on the video output of the ultrasound machine (e.g., 1280x1024, 1920x1080), the user can choose the right setting. On the right of the screen is the widget of the RAILUS software, where the model provides continuous real-time prediction (as the green bar) of the class of corresponding clips. The user can enable or disable the model prediction. The clinician interacts with the laptop using the mouse to select the lung zones (12 lung zones) and labels. After choosing the label and submitting, the lung zone in the lung diagram changes color (e.g. from green-normal lung to yellow-pleural effusion) according to the class of the label. For the purpose of testing and comparison between clinicians with the AI tool and without the AI tool, the bottom right shows the user prediction, model’s prediction, user’s confidence and total time taken for interpretation. When the user finishes the lung ultrasound examination, the tool will generate the result in the text file and the corresponding clips will be saved to the same folder.


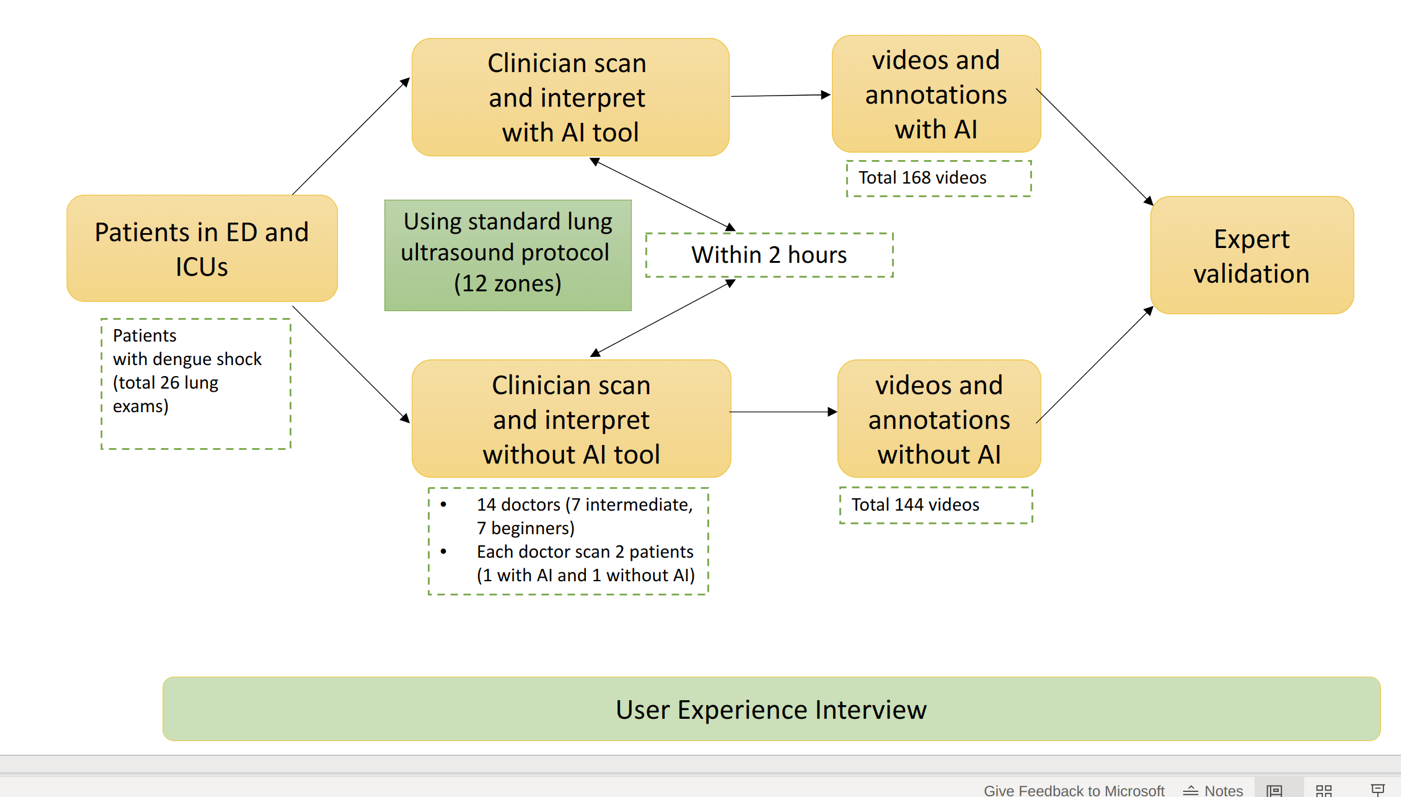


Fig. S5. Prospective study schema. Patients admitted to Emergency Department (ED) or ICU were recruited to the study after stabilization. The patients were scanned twice: first, by a clinician with the AI–assisted tool (RAILUS), and second, by another clinician without the AI–assisted tool 2 hours later. The video and annotations of the collected LUS video were validated by an independent LUS expert.

Table S3. Baseline of characteristics of study patients

| Demographics (n=7) |  |
| --- | --- |
| Age (years) (median, IQR) | 19 (17–31) |
| Gender (male) | 5/7 |
| BMI | 22.4 (20.6–24.6) |
| Heart rate (beats/minute) | 100 (93–105) |
| Blood pressure (median, IQR) | |
| SBP (mmHg) | 105 (99–120) |
| DBP (mmHg) | 75 (70–85) |
| Respiratory distress with oxygen requirement | 3/7 |
| Respiratory rate (breaths/ min) | 20 (22–29) |
| Shock | 7/7 |
| Plasma leak | 7/7 |

Performance of 14 clinicians in second phase who participated in the third phase was shown in confusion matrices in Fig. S6.


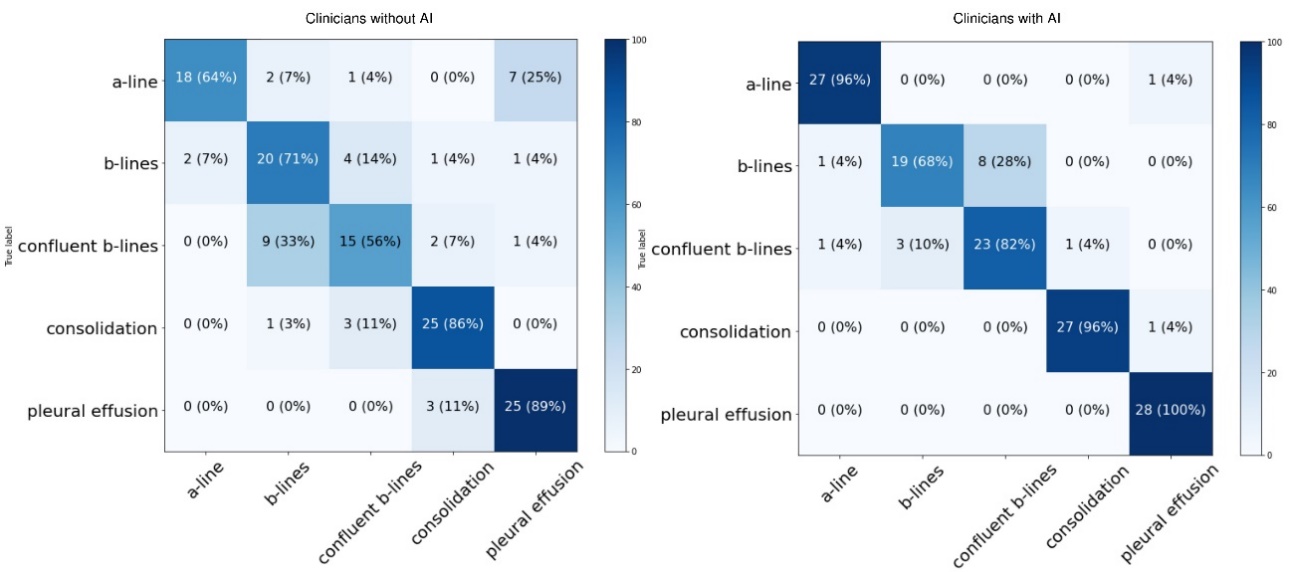


Fig S6. Performance of 14 clinicians in the third phase when using the tool offline in phase 2

# Section 3. Questionnaire - Usability of AI-assisted lung ultrasound interpretation tool

## Part 1 – Demographics (10 questions)

Table S4. Demographics of clinician’s questionnaires

| Level of training | 1. Ultrasound doctor 2. Intensivist 3. General Doctor 4. Resident 5. Student 6. Other |
| --- | --- |
| What is your experience in any ultrasound modality? | 1. Beginner 2. Intermediate 3. Advanced 4. Expert |
| What kind of ultrasound you have used and currently using? | 1. Cardiac 2. Abdominal 3. Blood vessel 4. Lung ultrasound 5. Intervention guidance 6. Other |
| What is your experience in lung ultrasound | 1. Beginner 2. Intermediate 3. Advanced 4. Expert |
| Is lung ultrasound available in your unit/apartment | 1. Yes 2. No |
| What do you think are the main challenges in doing lung ultrasound | 1. Finding the right view 2. Image interpretation 3. Image quantification 4. Identify landmark 5. Instruction for user 6. Physical environment 7. Writing report 8. Other |
| What kind of ultrasound machine you are currently using? | 1. Handheld devices (tablet, phone-based devices) 2. Point-of-care (ICU, ED) 3. “Full feature-trolley” machine |

## **Part 2 – Lung ultrasound video interpretation (10 standard videos and 10 videos with AI tool)**

All videos of the test can be found in this link: video_test_LUS

## Part 3 – Usability (20 questions)

The answers of the questionnaire were valued by five–point Likert scale. The questionnaire was developed and delivered by Google Forms. Because the interaction between AI-based tools and clinicians is poorly understood, the survey aimed at investigating how our LUS tool should be deployed in clinical settings, the clinician’s trust in the tool, and the level of concern about its utilisation.

Table S5. Usability questionnaire

| **Usability questionnaire** | **[1] Strongly disagree**  **-> [5] Strongly agree** | | | | |
| --- | --- | --- | --- | --- | --- |
|  | 1 | 2 | 3 | 4 | 5 |
| 1. I think that I would like to use this tool frequently |  |  |  |  |  |
| 2. I found the tool unnecessarily complex |  |  |  |  |  |
| 3. I thought the tool was easy to use |  |  |  |  |  |
| 4. I think that I would need the support of a technical person to be able to use this tool |  |  |  |  |  |
| 5. I found the various functions in this tool were well integrated |  |  |  |  |  |
| 6.I thought there was too much inconsistency in this tool |  |  |  |  |  |
| 7. I would imagine that most people would learn to use this tool very quickly |  |  |  |  |  |
| 8. I found the tool very cumbersome to use |  |  |  |  |  |
| 9. I felt very confident using the tool |  |  |  |  |  |
| 10. I needed to learn a lot of things before I could get going with the tool |  |  |  |  |  |
| 11. I feel that such a tool may improve the differential diagnostic process in the ICU |  |  |  |  |  |
| 12. To what extent do you agree with the following statement: “Seeing the model prediction bar will improve your confidence in interpret lung ultrasound videos” |  |  |  |  |  |
| 13. To what extent do you agree with the following statement: “The model prediction bar distract me from interpret lung ultrasound videos” |  |  |  |  |  |
| 14. To what extent do you agree with the following statement: “Artificial intelligence assisted tool can help improving quality of lung ultrasound” |  |  |  |  |  |
| 15. Have you used other AI-assisted tools before? If yes, is it useful?   - Yes - No | | | | | |
| 16. When do you think the tool will be most useful?   - Real-time (when carrying out the exam) - Offline (collect the video and interpret afterward) - Both | | | | | |
| 17. What level of error is acceptable for AI-assisted LUS interpretation in that are used for the patients?   - Equivalent to the worst performing advanced user - Equivalent to the average performing advanced user - Superior to the average performing advanced user - Equivalent to the best performing advanced user - Superior to the best performing advanced user | | | | | |
| 18. Would you consider using the following clinical workflow? Patients clinical images undergo AI-assisted LUS interpretation tool. A specialist subsequently reviews both the image and the artificial intelligence findings.   - Yes - No | | | | | |
| 19. Which of the following do you perceive as the greatest potential advantage of the use AI-assisted LUS interpretation tool? (rank the top 3 preferences where 1=greatest advantage)   - Assist non-expert clinician to carry out acceptable quality lung ultrasound - Improved patient access to disease screening - Improved diagnostic confidence - Reduced time spent by specialists on monotonous tasks - Greater uniformity in diagnosis and management decisions - Improved prediction of disease outcomes - People with less expertise can collect acceptable quality lung ultrasound examination - Other | | | | | |
| 20. Which of the following do you perceive as the concern to the utilisation of AI-assisted LUS interpretation tool? (rank the top 3 preferences where 1=greatest concerns)   - Concerns over the divestment of health care to large technology and data companies - Data security & privacy concerns - Concerns over medical liability due to machine error - Lack of confidence or trust in 'black-box' diagnosis - Decreasing reliance on medical specialists for diagnosis and treatment advice - Challenge to the fiduciary relationship between patient and doctor - Concerns over benchmarking clinicians against machines - Impact on workforce needs - Other | | | | | |

Table S6. Demographics of the participants. Detail of distribution of participants according to training, expertise and previous US experience

|  | **Phase 1**  **n = 276 (%)** | **Phase 2**  **n = 57 (%)** | **Phase 3**  **n = 14 (%)** |
| --- | --- | --- | --- |
| **Level of training** | |  |  |
| Radiology doctor | 105 (38%) | 6 (11%) | 4 (29%) |
| Intensivist | 57 (21%) | 15 (26%) | 3 (21%) |
| Doctor | 77 (28%) | 29 (51%) | 4 (29% |
| Resident | 20 (7%) | 2 (4%) | 1 (7%) |
| Other | 17 (6%) | 5 (9%) | 2 (14%) |
| **Experience in lung ultrasound** | |  |  |
| Beginner | 194 (70%) | 47 (82%) | 9 (64%) |
| Intermediate | 71 (26%) | 9 (16%) | 5 (36%) |
| Advanced | 7 (3%) | 1 (2%) | 0 (0%) |
| Expert | 4 (1%) | 0 (0%) | 0 (0%) |

**Table S7. Principal challenges in conducting lung ultrasound in clinical practice identified by participating clinicians**

| **Principal challenges in lung ultrasound** | |
| --- | --- |
| Image interpretation | 200 (72%) |
| Identifying landmark | 139 (50%) |
| Finding the right view | 137 (50%) |
| Writing report | 66 (24%) |
| Instruction for user | 78 (28%) |
| Image quantification | 58 (21%) |
| **Experience in general ultrasound** | |
| Beginner | 121 (44%) |
| Intermediate | 121 (44%) |
| Advanced | 30 (11%) |
| Expert | 4 (1%) |
| **Kind of ultrasound exam** | |
| Echocardiography | 115 (42%) |
| Abdomen | 178 (64%) |
| Blood vessel | 90 (33%) |
| Lung | 117 (42%) |
| Intervention guidance | 117 (42%) |
| Other | 18 (7%) |
| **Is lung ultrasound used in your unit/department** | |
| Yes | 177 (64%) |
| No | 99 (36%) |
| **Type of ultrasound machine available** | |
| Handheld devices (phone/tablet-based) | 10 (4%) |
| Point-of-care | 169 (61%) |
| "Full feature-trolley" big machine | 156 (57%) |


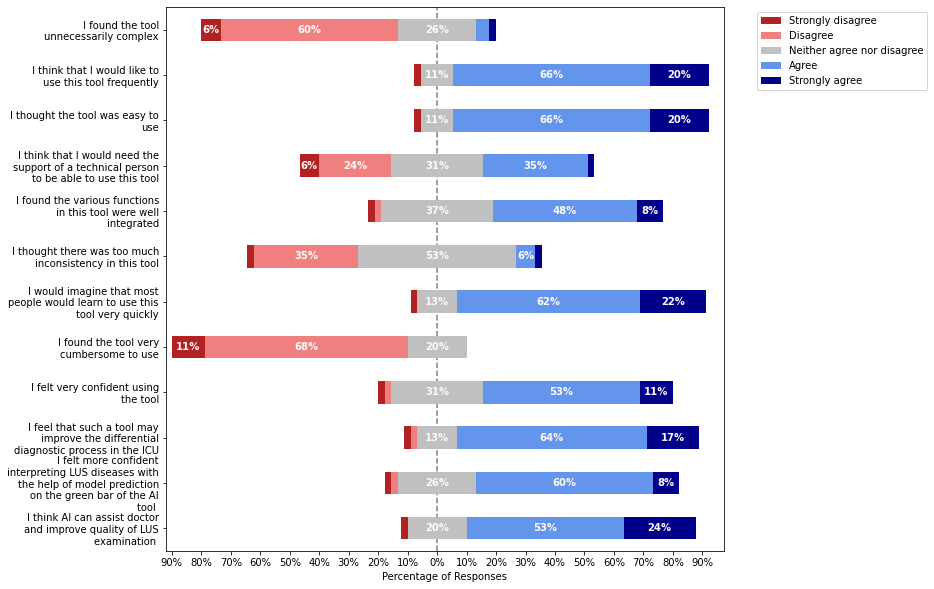


Fig. S7. Usability of the RAILUS software in real-time, from a survey carried out after operators performed LUS without and with RAILUS.

# References

1 Lazzeri C, Peris A. The Kigali modification of the berlin definition: A new epidemiological tool for ARDS? *J Thorac Dis* 2016; **8**: E443–5.

2 Dutta A, Zisserman A. The VIA Annotation Software for Images, Audio and Video. In: Proceedings of the 27th ACM International Conference on Multimedia. New York, NY, USA: ACM, 2019. DOI:10.1145/3343031.3350535.

3 Kerdegari H, Phung NTH, McBride A, *et al.* B-line detection and localization in lung ultrasound videos using spatiotemporal attention. *Appl Sci* 2021; **11**. DOI:10.3390/app112411697.
